# Computational Insight Into the Small Molecule Intervening PD-L1 Dimerization and the Potential Structure-Activity Relationship

**DOI:** 10.3389/fchem.2019.00764

**Published:** 2019-11-12

**Authors:** Danfeng Shi, Xiaoli An, Qifeng Bai, Zhitong Bing, Shuangyan Zhou, Huanxiang Liu, Xiaojun Yao

**Affiliations:** ^1^State Key Laboratory of Applied Organic Chemistry, Department of Chemistry, Lanzhou University, Lanzhou, China; ^2^School of Basic Medical Science, Lanzhou University, Lanzhou, China; ^3^Institute of Modern Physics of Chinese Academy of Sciences, Lanzhou, China; ^4^School of Pharmacy, Lanzhou University, Lanzhou, China; ^5^State Key Laboratory of Quality Research in Chinese Medicine, Macau Institute for Applied Research in Medicine and Health, Macau University of Science and Technology, Macau, China

**Keywords:** PD-L1, small-molecule inhibitors, molecular dynamics simulation, metadynamics simulation, R-group QSAR

## Abstract

Recently, small-molecule compounds have been reported to block the PD-1/PD-L1 interaction by inducing the dimerization of PD-L1. All these inhibitors had a common scaffold and interacted with the cavity formed by two PD-L1 monomers. This special interactive mode provided clues for the structure-based drug design, however, also showed limitations for the discovery of small-molecule inhibitors with new scaffolds. In this study, we revealed the structure-activity relationship of the current small-molecule inhibitors targeting dimerization of PD-L1 by predicting their binding and unbinding mechanism via conventional molecular dynamics and metadynamics simulation. During the binding process, the representative inhibitors (BMS-8 and BMS-1166) tended to have a more stable binding mode with one PD-L1 monomer than the other and the small-molecule inducing PD-L1 dimerization was further stabilized by the non-polar interaction of Ile54, Tyr56, Met115, Ala121, and Tyr123 on both monomers and the water bridges involved in _A_Lys124. The unbinding process prediction showed that the PD-L1 dimerization kept stable upon the dissociation of ligands. It's indicated that the formation and stability of the small-molecule inducing PD-L1 dimerization was the key factor for the inhibitory activities of these ligands. The contact analysis, R-group based quantitative structure-activity relationship (QSAR) analysis and molecular docking further suggested that each attachment point on the core scaffold of ligands had a specific preference for pharmacophore elements when improving the inhibitory activities by structural modifications. Taken together, the results in this study could guide the structural optimization and the further discovery of novel small-molecule inhibitors targeting PD-L1.

## Introduction

The blockage of the protein-protein interaction (PPI) between programmed cell death protein 1 (PD-1) and programmed cell death 1 ligand 1 (PD-L1) can reactivate the effector functions of T cell and eliminate tumor phenotypes with significant PD-L1 expression (Gatalica et al., 2014; Patel and Kurzrock, 2015; Sharma and Allison, 2015a,b). The crystal structures of PD-1/PD-L1 complex revealed the interface and hot-spot domains for both proteins (Zak et al., 2015; Pascolutti et al., 2016), which provided the structural basis for drug design. Ligands such as monoclonal antibodies (mAbs) (Lee et al., 2016, 2017; Liu K. et al., 2016; Tan et al., 2017; Zhang et al., 2017a,b), peptides (Chang et al., 2015; Magiera-Mularz et al., 2017), and small-molecule compounds (Abdel-Magid, 2015; Zak et al., 2016; Skalniak et al., 2017) had been discovered to interact with the PPI interface of PD-1 or PD-L1, showed obvious inhibitory activities against PD-1/PD-L1 signaling pathways. As the small-molecule inhibitors have better characteristic on aspects like production cost, drug-like property, immunogenic side effects, and half-life period (Liu K. et al., 2016) than peptides and monoclonal antibodies, the development of small-molecule inhibitor tended to be more promising. The crystal structures of small-molecule complex provided a good chance for the drug design of anti-cancer immunotherapy targeting on PD-1/PD-L1 immune checkpoint.

According the patents by Bristol-Myers Squibb (BMS) company, the compounds with (2-methyl-3-biphenylyl)methanol scaffold were privileged for inducing the dimerization of PD-L1 and interacted with the hydrophobic tunnel formed by two PD-L1 monomers (Zak et al., 2016; Guzik et al., 2017). Previously, George F. Gao's group resolved a dimeric interface of PD-L1 formed by B, C″, D, and E strands on each monomer, which was proved to be either a functional unit in immunological synapse formation or a revolution relics of B7 family (Tan et al., 2016). The crystal lattice analysis by Zak et al. also didn't suggest the spontaneous dimerization of PD-L1 (Zak et al., 2015), indicating that the interfacial interaction between two PD-L1 monomers was quite weak for dimerization process. As for the small molecule intervening PD-L1 dimerization, the interacting interface analysis showed that these ligands interacted with the G, F, C, C′ strands of PD-L1 in a competitive manner vs. PD-1 like mAbs or peptide inhibitors (Sharpe et al., 2011; Liu A. et al., 2016). Specially, the dimerized crystal structures tend to be a common pharmacodynamic characteristics for BMS small-molecule analogs despite of inhibitory activity difference from millimole to nanomole level (Abdel-Magid, 2015; Zak et al., 2016; Skalniak et al., 2017; Perry et al., 2019). Considering the potential relationship between the inhibitory activities of BMS small-molecule inhibitors and the stabilities of the dimerized complex systems, the dimerization process and the structure-activity relationship of small-molecule inhibitors need to be further elucidated. Besides, the broad, scattered and hydrophobic interface on PD-L1 makes it difficult for the discovery of novel small molecule ligands and also results in the strong hydrophobicity of BMS small-molecule inhibitors (Zarganes-Tzitzikas et al., 2016). Therefore, an understanding of the inhibitory mechanism of small-molecule ligands targeting PD-L1 such as key residues at the binding site, effect of the solvation and binding or unbinding process of small molecule inhibitors would help in the discovery of novel inhibitors and structural optimization of reported small-molecule inhibitors.

In this study, we aimed to reveal the detailed molecular mechanism of BMS small-molecule inhibitors from the formation and disassociation of PD-L1 dimers by multiple molecular modeling methods. Two representative compounds (BMS-8 and BMS-1166) with known inhibitory activities and complex crystal structures were selected to perform molecular dynamics simulations. During the formation process, both monomer and dimer systems of PD-L1 in complex with small-molecule ligands were applied to evaluate the stabilities of binding modes between ligands and PD-L1. The binding free energy calculation by MM-PBSA and MM-GBSA (Genheden and Ryde, 2015; Chen et al., 2018; Sun et al., 2018) were also used to analyze the energy contribution of the interfacial residues on PD-L1 dimers. During the disassociation process, metadynamics simulations (Bernardi et al., 2015) with specific collective variables (CVs) were performed to explore the key transition states along unbinding pathways. Based on the results of molecular modeling, an interplay mechanism of BMS small-molecule ligands with PD-L1 was proposed. Finally, R-group based QSAR analysis (Holliday et al., 2003; Hirons et al., 2005) and molecular docking were constructed on the reported BMS small-molecule inhibitors. The results of this study would provide a good guidance for the discovery of novel small-molecule inhibitors and structural modification of BMS small-molecule inhibitors targeting PD-L1.

## Methods and Materials

### The Conventional Molecular Dynamics Simulations

The complex crystal structures of BMS-8 and BMS-1166 were used to perform conventional molecular dynamics simulations. The Cartesian coordinates of the heavy atoms of PD-L1 (sequence 18–132) and small-molecule ligands were derived from the PDB database with accession number of 5J8O (Zak et al., 2016) and 5NIX (Skalniak et al., 2017). In order to eliminate the electrostatic effect of terminal residues, both monomers were capped with ACE and NME at two ends. The simulation details of the monomer systems and dimer systems were shown in Table 1. All the complex systems were firstly prepared through structural inspection and optimization in Schrödinger 2015 software suite (Schrödinger, LLC: New York, NY, 2015). Then, the complex proteins were solvated in a rectangular box of TIP3P waters and neutralized with Na^+^ ions. The periodic boundary conditions were setup with all the solvents at least 10 Å away from the complex. Then, the solvated systems were parameterized using the AMBER FF14SB force field (Case et al., 2014). The molecular dynamics simulations were performed in four steps. Firstly, energy minimization was performed to remove the local atomic collision in the systems. The energy minimization was conducted by both descent steepest method and conjugated gradient method with 5,000 steps. Then, the temperature of each system was gradually upgraded from 0 to 300 K in the NVT ensemble with all the solute atoms constrained with a force constant of 2.0 kcal mol^−1^·Å^−2^. After that, each system was equilibrated with the force constant decreasing from 2.0 to 0 kcal mol^−1^·Å^−2^ in a period of 1 ns. Finally, a production run of 150 ns was performed for each system in the NPT ensemble at 300 K and 1.0 atm condition. The snapshots for all the trajectories were saved every 2 ps.

**Table 1:** The details of conventional molecular dynamics simulations.

	**Dimer systems**	**Monomer systems**	
	**PD-L1 dimer**	**PD-L1 (Conformation A)**	**PD-L1 (Conformation B)**
BMS-8	150 ns	150 ns × 2	150 ns × 2
BMS-1166	150 ns	150 ns × 2	150 ns × 2

### The Binding Free Energy Calculation

For dimer systems of BMS-8 and BMS1166, two PD-L1 monomers were selected as the receptor, while small-molecule inhibitors were selected as the ligand. Both MM-PBSA and MM-GBSA methods were performed to calculate the binding free energy of BMS inhibitors according to the equation below:

(1) $$\begin{array}{l} \Delta G=<G_{Complex}-G_{Receptor}-G_{Ligand}> \end{array}$$

Where < > represents the average value for all the snapshots used for MM-PBSA and MM-GBSA calculation. Different energy terms can be estimated as follows:

(2) $$\begin{array}{l} \Delta G=\Delta H-T\Delta S \end{array}$$

(3) $$\begin{array}{l} \Delta H=\Delta E_{gas}+\;\Delta E_{sol}\;=\Delta E_{polar}+\;\Delta E_{nonpolar} \end{array}$$

(4) $$\begin{array}{l} \Delta E_{gas}=\Delta E_{int}+\;\Delta E_{ele}+\;\Delta E_{vdW} \end{array}$$

(5) $$\begin{array}{l} \Delta H_{sol}=\Delta E_{ele,\;sol}+\;\Delta E_{nonpl,sol} \end{array}$$

(6) $$\begin{array}{l} \Delta E_{nonpl,sol}=\gamma *\Delta SASA \end{array}$$

500 snapshots were extracted from the last 20 ns trajectories and used for MM-PBSA and MM-GBSA calculation. The parameter settings during MM-PBSA and MM-GBSA calculation were referred to the previous works published by our group (Xue et al., 2013). Then, the per-residue based decomposition was performed to identify the key residues in both dimer systems. Finally, the contribution of entropy change (–*T*Δ*S*) was calculated by 100 snapshots from the last 20 ns trajectory.

### The Calculation of Water Occupancies

The water molecules on the surface affected the conformational stability of proteins (Bellissent-Funel et al., 2016). By calculating the water occupancies on the surface of protein complex, water sites with a higher probability of finding a water molecule could be identified (Gauto et al., 2013). The water molecules at those sites were involved in the water bridges between protein and ligand and could enhance the stability of protein complex thermodynamically (Romero et al., 2016). To evaluate the effects of the water-mediated complex stability upon the binding of BMS inhibitors, the water occupancies and the water bridges were calculated over the last 20 ns trajectories for each dimer system using the “cpptraj” module of the AMBER14. All the trajectories were first imaged and fit to the first frame by the root mean square deviation (RMSD) of the heavy atoms of PD-L1 dimers. Then, the water occupancies were calculated using the “grid” command with a 0.5 Å ^*^0.5 Å ^*^ 0.5 Å spacing over the whole box. And the water occupancies for both dimer systems were represented in the Chimera software (Pettersen et al., 2004).

### Metadynamics Simulations

Metadynamics simulations have been widely used to predict the unbinding pathways and dissociation energy barrier of ligands for ligand-target systems (Cavalli et al., 2015). The sampling process of metadynamics simulations had an advantage of not requiring an initial estimate of the energy landscape to explore by periodically adding history-dependent biasing potential on selected collective variables (CVs) (Masetti et al., 2009; Barducci et al., 2011; Casasnovas et al., 2017; Sun et al., 2017). In this study, CV1 was selected as the distance between the mass center of the heavy atoms on ligand and the mass center of heavy atoms on key residues including Ile54, Tyr56, Met115, Ala121, Tyr123 in both chains; CV2 was selected as the angle between the C_α_ atom of Tyr56 and two carbon atoms that were the furthest away from each other on the core scaffold. The metadynamics simulations were performed for both dimer systems. The prepared topology files and coordinate files by AMBER ff14SB force field were further applied in the NAMD2.9 software (Kalé et al., 1999) implemented by PLUMED code (Bonomi et al., 2009). The initial structures were minimized for 5,000 steps with all the atoms on protein and ligand restrained with 5 kcal mol^−1^·Å^−2^ and all restraints released therewith. Then the temperature of systems were upgraded to 300 K in 30,000 steps. Afterward, all the systems were submitted to two short time NVT simulations (100,000 steps) to equilibrate the systems with restraining force constant of 5 kcal mol^−1^·Å^−2^ and all restraints released therewith. Finally, the equilibrated structures restarted from the NVT simulation were used for metadynamics simulations.

(7) $$\begin{array}{l} V\left(s,t\right)\;=\underset{k\tau \;<\;t}{\sum }W\left(k\tau \right)\;exp\left(-\overset{d}{\underset{i=1}{\sum }}\frac{{\left(s_{i}-s_{i}\left(q\left(k\tau \right)\right)\right)}^{2}}{2\sigma_{i}^{2}}\right) \end{array}$$

(8) $$\begin{array}{l} W\left(k\tau \right)\;=W_{0}\;exp\left(-\frac{V\left(s\left(q\left(k\tau \right)\right)\right),k\tau }{K_{B}\Delta T}\right) \end{array}$$

(9) $$\begin{array}{l} \gamma =\frac{T+\;\Delta T}{T} \end{array}$$

Metadynamics could reconstruct the free-energy surface as a function of specific collective variables (CVs). The general formalism of history-dependent Gaussian potential was shown as Equation (7). *V* represents the sum of the history-dependent Gaussian potential along the specific reactive coordinate (*s*_*i*_) during time span (*k*τ). In this study, the deposition time (τ) was set as 1 ps to give enough dissociation time for ligands without adding biasing potential on the dissociation boundary. The Gaussian width (σ) of CV1 and CV2 were set to 0.8 Å and 0.02 rad, respectively. As for the well-tempered metadynamics, the height of the Gaussian potential (*W*) is affected by a parameter Δ*T* as Equation (8). The initial hill height (*W*_0_) of Gaussian potential was set to 0.6 kcal/mol·ps and the bias-factor (γ) was set to 10 with a temperature (*T*) of 300 K to control the decrease rate of the biasing potential as Equation (9).

### R-Group QSAR Model and Molecular Docking of BMS Small-Molecule Inhibitors

The pharma R-group quantitative structure-activity relationship (RQSAR) models tended to be an effective approach for the SAR evaluation of the congeneric series of compounds (Adhikari et al., 2015). It was more suggestive than other approaches for the structural modification of small-molecule inhibitors by identifying the core scaffold and evaluating the effective pharma element at different attachment points (Kolarevic et al., 2018; Ts Mavrova et al., 2018). A total of 110 BMS small-molecule inhibitors with 2-methyl-3-(phenoxymethyl)-1,1'-biphenyl scaffold were collected from the patents of BMS company (Abdel-Magid, 2015; Table S1). All these small molecules had seven attachment points and diverse substitution groups, which were suitable to perform R-group QSAR analysis in the Canvas software of Schrödinger Suite (Duan et al., 2010; Sastry et al., 2010). The linear relationship between the substitutions and the activities (–log IC_50_) was analyzed and the importance of six key pharmacophore elements including hydrogen bond acceptor (A), hydrogen bond donor (D), hydrophobic group (H), negative ionic group (N), positive ionic group (P), and aromatic ring (A) were evaluated at each attachment point. During the process, the error and the importance were both set as 0.30. Eight representative small-molecule inhibitors (NO. of compound:4, 101, 102, 103, 104, 108, 109, 110) with substitutions on R1, R2, or R3 were selected to perform molecular docking to further study the binding modes. In order the compare the effect of R-groups, the core scaffold atoms with SMILES of “cOCc(c1C)cccc1c” were constrained with RMSD of 0.5 angstrom while other atoms were selected flexible. The standard precision (SP) docking score was used to evaluate the binding poses. The molecular docking was performed in Schrödinger 2015 software suite (Schrödinger, LLC: New York, NY, 2015).

### Residue-Ligand Contact Analysis

In this study, we performed residue-ligand contact analysis to detect the surrounding residues around different substituent groups of BMS-8 and BMS-1166. It is assumed that the contacts exist between two groups as long as their distance was below a cutoff of 3.5 Å. The occupancy of each contact was calculated by the existence frequency in the 5,000 snapshots of the last 50 ns trajectories.

## Results and Discussion

### The Conformational Stabilities Between PD-L1 Monomer and BMS Small-Molecule Inhibitors

**Figure 1:**
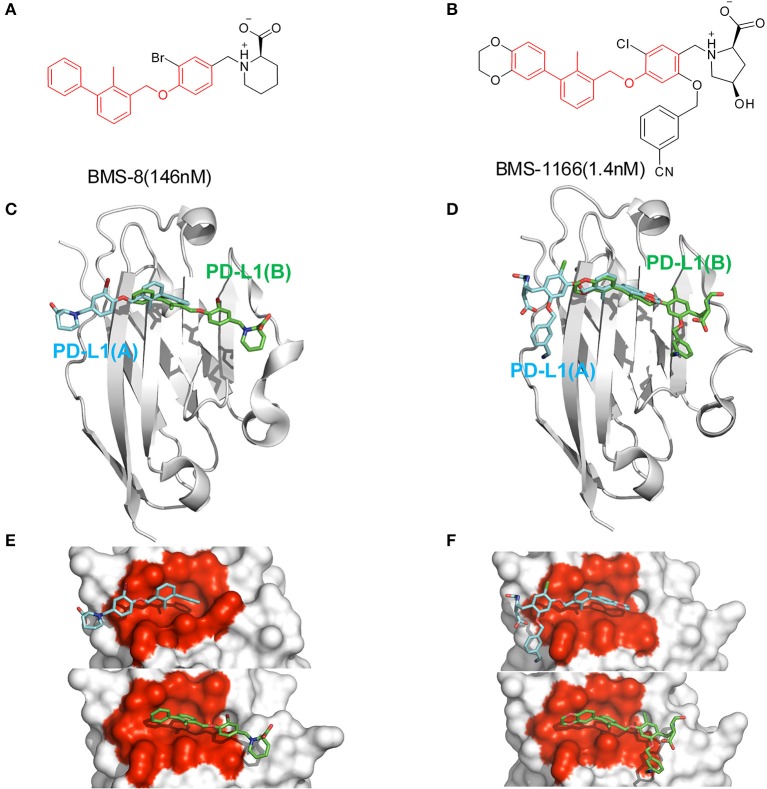
The structural information of BMS-8 and BMS-1166. **(A,B)** The chemical formulas of BMS-8 and BMS-1166. The core scaffold is colored in red. **(C,D)** The conformational superposition of BMS-8 and BMS-1166 interacting with the monomer conformation A, B of PD-L1. **(E,F)** The surface of PD-L1 (A) and PD-L1 (B) interacting with BMS-8 and BMS-1166. The binding pockets formed by I54, V55, Y56, M115, I116, S117, A121, D122, and Y123 were colored in red.

**Figure 2:**
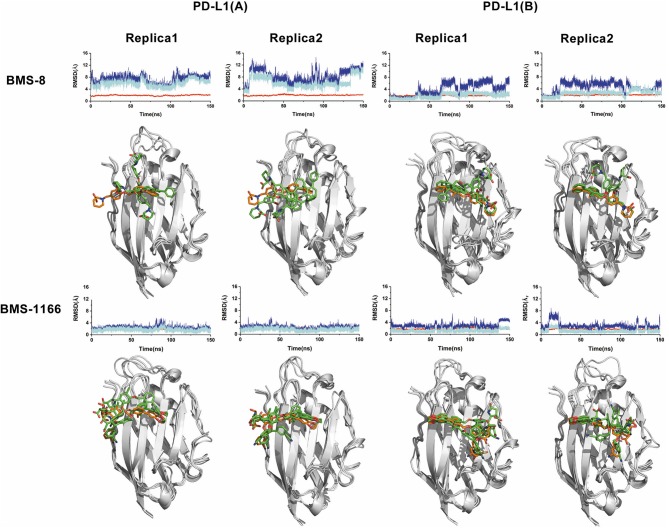
The stability evaluation of the monomer systems. The RMSDs of the heavy atoms of PD-L1 monomer, ligands (BMS-8, BMS-1166) and the core scaffold of the ligand are shown in red, blue, and cyan lines, respectively. The representative conformations of three clusters for every monomer system was shown below the corresponding system. PD-L1 is shown in gray cartoon while the initial conformation of ligand is shown in orange sticks and the dynamics conformations of ligand is shown in green sticks.

In order to explore the interactive process of BMS small-molecule inhibitors, we constructed two kinds of ligand-bound PD-L1 monomer systems as shown in Figure 1 and used molecular dynamics simulations to evaluate the stabilities of both binding modes by two replicas. As shown in Figure 2, the stabilities of both binding modes were evaluated by the RMSD of the heavy atoms of receptor, ligand and the core scaffold of ligands in two replicas. The core scaffold of BMS-8 tended to have a more stable contact with the conformation B than conformation A of PD-L1 according to the comparison of RMSD and the representative conformations of both binding modes. The detailed docking interactions diagram in Figures S1, S2 showed that the π-π stacking interaction between the biphenyl moiety and _A_Tyr56 tended to be easily affected by the conformation of _A_Tyr56 and unstable among three clusters, while the hydrophobic interaction between biphenyl moiety and residues on conformation B of PD-L1 tended to be stable among all three clusters. As for BMS-1166, both binding modes seemed to be quite stable, which probably accounting for the best inhibitory activities of BMS-1166 among the small-molecule inhibitors of BMS. The detailed docking interactions diagram in Figures S3, S4 showed that the biphenyl moiety had less conformational fluctuation and more stable hydrophobic interactions among the clusters of conformation A and B of PD-L1. The stabilities of the monomer complex of PD-L1 and ligand was affected by the hydrophobic interactions and turned out to be associated with the inhibitory activities of BMS small-molecule inhibitors.

### The Interaction Stabilities Between PD-L1 Dimer and BMS Small-Molecule Inhibitors

**Figure 3:**
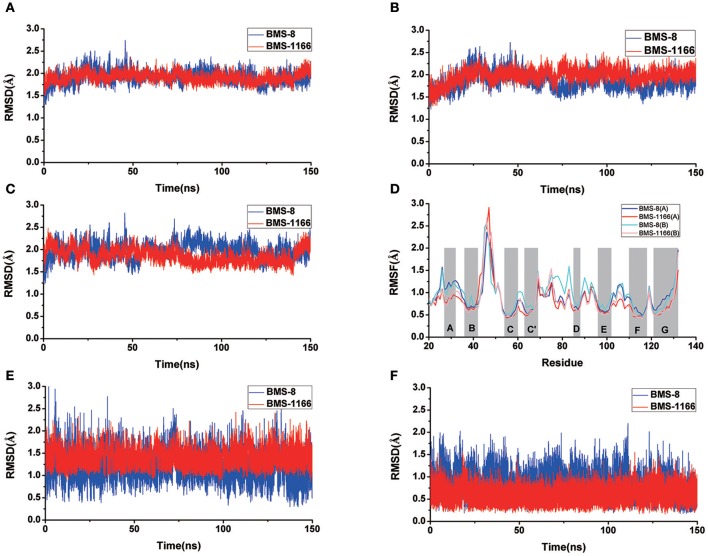
The RMSDs and RMSFs of the heavy atoms in dimer systems. **(A)** RMSD of complex, **(B)** RMSD of PD-L1 at conformation A, **(C)** RMSD of PD-L1 at conformation B, **(D)** RMSF of residues on PD-L1 at conformation A, B, **(E)** RMSD of the ligand, and **(F)** RMSD of the core scaffold.

The conformational stabilities of the dimer systems were evaluated by root mean square deviation (RMSD) and mean square root fluctuation (RMSF) as shown in Figure 3. The RMSDs of the complex, two PD-L1 monomers in complex systems showed that both dimer and monomers of PD-L1 had strong structural stabilities upon ligand binding. The conformational fluctuation of PD-L1 indicated that PD-L1 showed more flexibilities upon BMS-8 binding than BMS-1166. The comparison of the RMSDs of the core scaffold of two ligands showed that BMS-1166 had a more stable binding modes than BMS-8. The binding free energies were also calculated to evaluate the affinities of dimer systems. As shown in Table 2, the energy items of ΔΔG_PB_, ΔΔG_GB_, and ΔΔE_exp_ by MM-PBSA and MM-GBSA methods could properly evaluate the difference of affinities of BMS-8 and BMS-1166, which showed the fact that the affinities between small-molecule inhibitors and PD-L1 dimer could reflect the inhibitory activities relatively. BMS-1166 had a stronger enthalpy contribution (ΔΔH_PB_, ΔΔH_GB_) and a worse entropy contribution (–TΔΔS) than BMS-8, which were consistent with the stability difference of BMS-8 and BMS-1166 dimer complex.

**Table 2:** The binding free energies of BMS-8 and BMS-1166 evaluated by MM-PBSA, MM-GBSA, and metadynamics simulations.

**Contribution^**a**^**	**BMS-8**	**BMS-1166**	**ΔΔE (ΔE_**BMS−1166**_ – ΔE_**BMS−8**_)**
ΔE_ele,gas_	−29.29 ± 6.14	−72.23 ± 12.05	−42.94 ± 13.52
ΔE_vdw,gas_	−63.45 ± 2.77	−81.60 ± 3.72	−18.15 ± 4.64
ΔE_nonpl,sol_	−6.62 ± 0.18	−9.17 ± 0.19	−2.55 ± 0.26
ΔE_polar,sol,PB_	49.89 ± 7.19	105.28 ± 10.16	55.39 ± 12.45
ΔE_sol,PB_	43.27 ± 7.07	96.11 ± 10.11	52.84 ± 12.34
ΔE_polar,sol,GB_	45.04 ± 5.85	93.32 ± 9.91	48.28 ± 11.51
ΔE_sol,GB_	38.42 ± 5.75	84.15 ± 9.88	45.73 ± 11.43
ΔH_PB_	−49.49 ± 3.71	−57.72 ± 4.64	−8.23 ± 5.94
ΔH_GB_	−54.32 ± 3.14	−69.69 ± 3.32	−15.37 ± 4.57
–TΔS	24.72 ± 5.75	27.02 ± 5.89	2.30 ± 8.23
ΔG_PB_	−24.77 ± 6.84	−30.70 ± 7.50	−5.93 ± 10.15
ΔG_GB_	−29.60 ± 6.55	−42.67 ± 6.76	−13.07 ± 9.41
ΔE_CV1_^b^	−16.23	−28.79	−12.56
ΔE_CV2_^c^	−15.51	−27.89	−12.38
ΔE_exp_^d^	−9.32	−12.07	−2.75

a *The unit for the free energy contributions are shown in kcal/mol*.

b, c Δ*E_CV1_ and* Δ*E_CV2_ were estimated by the history-dependent free energy surfaces along CV1 and CV2*.

d *The experimental affinities for BMS-8 and BMS-1166 were extracted from the reference and calculated by using the equation as follows:* Δ*G = −RTln(1/IC_50_) at 298.15 K*.

**Figure 4:**
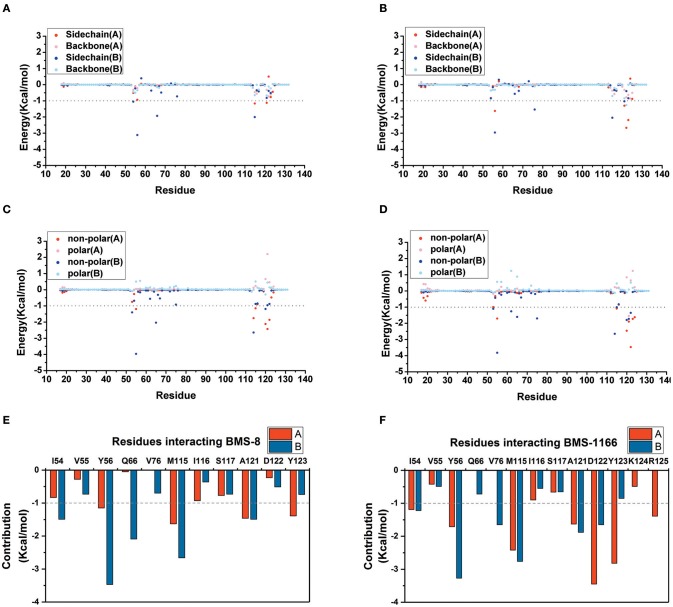
The residue energy decomposition of the key residues for BMS-8 **(A,C,E)** and BMS-1166 **(B,D,F)** dimer systems. **(A,B)** The energy contribution of sidechain and backbone, **(C,D)** the energy contribution of polar and non-polar interaction, **(E,F)** the total energy contribution for each residue. The cutoff value of the energy contribution for key residues were set as −1 kcal/mol.

**Figure 5:**
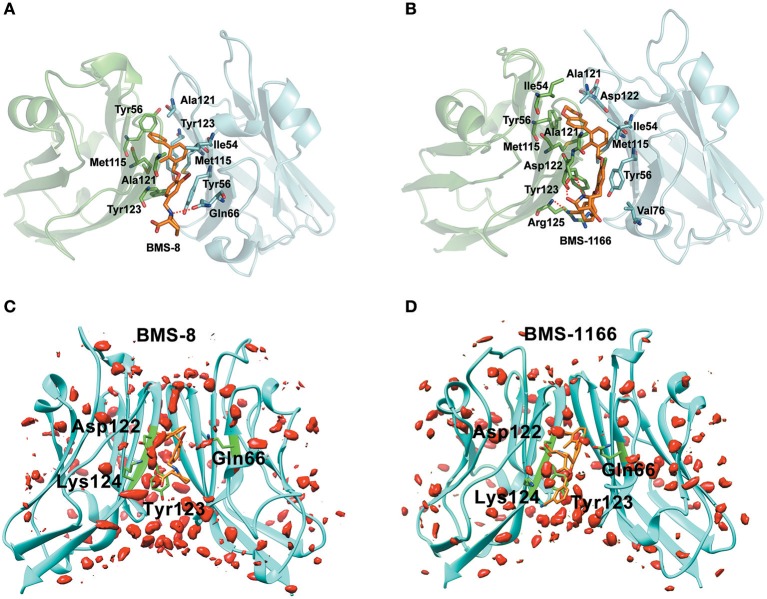
The binding modes and water occupancies in dimer systems. **(A,B)** The binding modes of BMS-8 and BMS-1166. The key residues were shown in green and cyan sticks and the ligand was shown in orange sticks. The hydrogen bonds were shown in red dash. **(C,D)** The water occupancies in BMS-8 and BMS-1166 dimer systems. The PD-L1 dimer is shown in cyan cartoon and the residues involved in water bridges are shown in green sticks. The water distributions are shown in red solid surface and the small molecule ligands are shown in orange sticks.

The key residues on two PD-L1 monomers interacting with ligands were recognized by per-residue energy decomposition. The energy contribution for each residue were decomposed into the sidechain part and the backbone part, the non-polar part and the polar part as shown in Figures 4A–D. It could be seen that BMS-8 and BMS-1166 mainly formed non-polar interactions with the sidechain of the residues on PD-L1. With a cutoff value of −1.0 kcal/mol, the key residues in BMS-8 dimer system included _A_Tyr56, _A_Met115, _A_Ala121, _A_Tyr123 and _B_Ile54, _B_Tyr56, _B_Gln66, _B_Met115, _B_Ala121 as shown in Figure 4E, while the key residues in BMS-1166 dimer system included _A_Ile54, _A_Tyr56, _A_Met115, _A_Ala121, _A_Asp122, _A_Tyr123, _A_Arg125 and _B_Ile54, _B_Tyr56, _B_Val76, _B_Met115, _B_Ala121, _B_Asp122 as shown in Figure 4F. Taken together, the interaction residues on conformation A and conformation B of PD-L1 were symmetrical both including Ile54, Tyr56, Met115, Ala121, and Tyr123. The hydrogen bond analysis in Table 3, Figures 5A,B showed that the protonated tertiary ammonium in BMS-8 formed a hydrogen bond with the side-chain oxygen of _B_Gln66 with an occupancy of 57.21%, while the BMS-1166 dimer system also formed hydrogen bond between the ammonium group on BMS-1166 and the carboxyl group of _A_Asp122. The binding mode analysis of substitute groups on BMS-8 and BMS-1166 with the interfacial residues on PD-L1 indicated that the interaction with the peripheral residues including _A_Ile54, _A_Arg125, _B_Val76, and _B_Asp122 could significantly enhance the inhibitory activities of BMS small-molecule inhibitors.

**Table 3:** The hydrogen bond analysis of the BMS-8 and BMS-1166 dimer systems.

**Acceptor**	**DonorH**	**Donor**	**Occupancy^**a**^ (%)**	**Distance (Å)^**b**^**	**Angle (**°**)^**c**^**
_B_Gln66@OE1	BMS-8@H1	BMS-8@N1	57.21	2.90	162.74
_A_Asp122@OD2	BMS-1166@H1	BMS-1116@O5	63.54	2.71	161.10
_A_Asp122@OD1	BMS-1166@H1	BMS-1116@O5	47.58	2.93	151.41
_A_Asp122@OD1	BMS-1166@H20	BMS-1116@N2	41.43	2.93	144.20
_A_Asp122@OD2	BMS-1166@H20	BMS-1116@N2	29.74	3.01	140.98
_A_Asp122@OD1	BMS-1166@H19	BMS-1116@N2	21.02	2.90	139.00
BMS-1166@N1	_A_Arg125@H	_A_Arg125@N	81.70	3.08	149.17
BMS-1166@O5	_A_Thr20@HG1	_A_Thr20@OG1	36.64	2.95	154.22

a *The occupancy of hydrogen bonds were analyzed through the last 20 ns trajectories and only hydrogen bonds with an occupancy more than 0.20 were extracted and shown*.

b, c *The hydrogen bonds were determined by an acceptor-donor atom distance of <3.5 Å and acceptor H-donor angle of >120°*.

In order to analyze the effect of solvent on PD-L1 dimer complex, water occupancies and water bridges involved in receptor-ligand interaction were both calculated. As shown in Table 4, the residues or residue pairs involved in water bridges with an occupancy higher than 20% were extracted from both dimer systems. It can be seen in Figure 5 that three water bridges involved in _A_Asp122, _A_Tyr123, _A_Lys124, and _B_Gln66 were stable in both dimer systems. Both ligands formed a strong water bridge with _A_Lys124 with an occupancy higher than 90%, which indicated that _A_Lys124 had a significant effect on the stabilities of the ligand conformations.

**Table 4:** The water bridge with occupancies higher than 20.00% in the BMS-8 and BMS-1166 dimer systems.

**Residues involving in water bridge**	**BMS-8 (%)**	**BMS-1166 (%)**
_A_Thr20	–	32.42
_A_Asp122	25.04	–
_A_Lys124	91.57	94.15
_A_Arg125	–	22.63
_B_Gln66	58.62	32.93
_B_Lys75	–	22.58
_B_Val76	–	50.13
_B_Asp122	–	29.66
_A_Phe19, _A_Ala121, _A_Asp122	–	23.52
_A_Asp122, _A_Tyr123, _A_Lys124	38.90	65.18

### The Disassociation Process of BMS Small-Molecule Inhibitors

**Figure 6:**
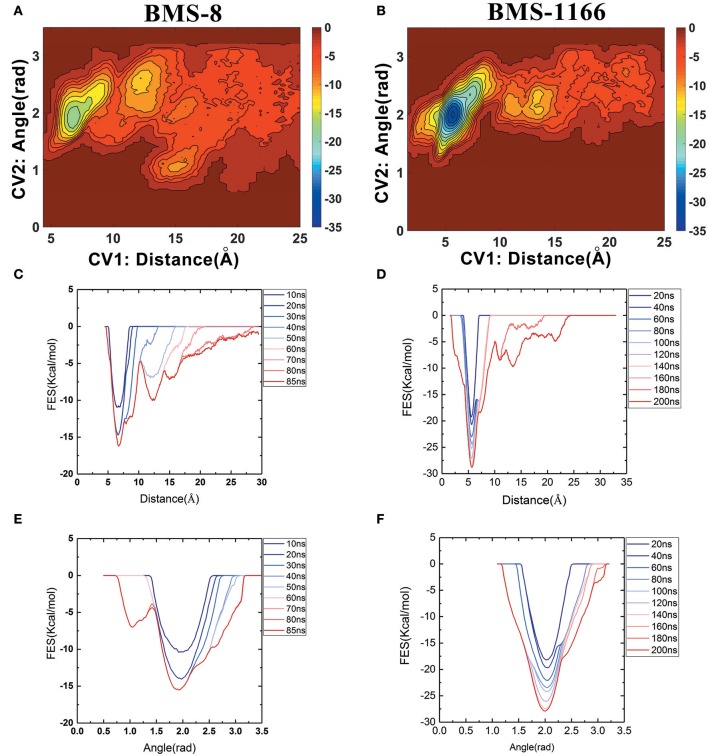
The energy change during the unbinding process. **(A,B)** The free energy landscapes for the unbinding process of BMS-8 and BMS-1166. **(C–F)** The convergence of sampling process during the unbinding process of BMS-8 and BMS-1166. The history-dependent free energy surfaces along CV1 **(C,D)** and CV2 **(E,F)** are estimated by a segmented accumulation of simulation time. The unit for the free energy is kcal/mol.

The free energy landscape of the unbinding processes of both BMS small-molecule inhibitors were constructed by CV1 and CV2. The distribution of minima in the landscapes showed that the most stable conformational state in the unbinding process was corresponding to the conformational states of the initial crystal structures as shown in Figures 6A,B. During the unbinding process, there were four different transition states for BMS-8 and three transition states for BMS-1166. In order to test the convergence of unbinding process, the free energies along both CVs were estimated. It can be seen that the free energy surface of CV1 (Figures 6C,D) and CV2 (Figures 6E,F) gradually came to a convergence along with the accumulation of time. As CV1 represented the distance between the ligand and the binding site of PD-L1 dimer and depicted the unbinding process better than CV2, the corresponding minimum points along CV1 were extracted from the unbinding trajectories. In BMS-8 complex systems, the minima along CV1 were 6.75 Å (−16.34 kcal/mol), 12.54 Å (−10.12 kcal/mol), and 14.97 Å (−7.31 kcal/mol). In BMS-1166 complex systems, the minima along CV1 were 5.67 Å (−28.79 kcal/mol), 10.95 Å (−8.50 kcal/mol), and 13.46 Å (−9.74 kcal/mol). The ultimate unbinding energy barriers of both small-molecule ligands estimated by CV1 and CV2 were shown in Table 1 and Figure 6, which were in good consistency with the inhibitory activities. Considering the difference between the binding free energies predicted by different methods, MM-PBSA and MM-GBSA calculated the binding free energies using implicit water models while metadynamics simulation considered the explicit water interaction between protein and ligand. Therefore, the results from metadynamics simulation tended to be more approximate to the experimental results.

**Figure 7:**
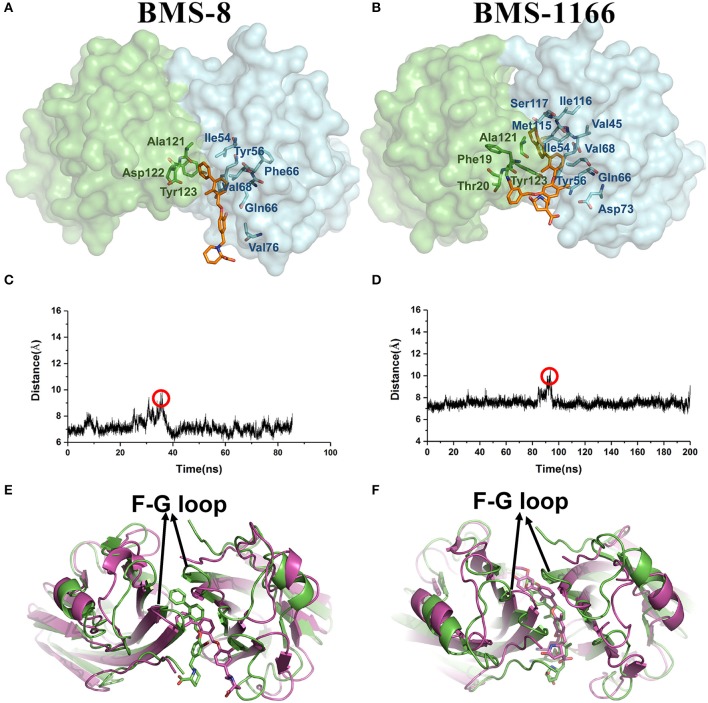
The conformational change during the unbinding process. **(A,B)** The key transition conformational states of BMS-8 and BMS-1166 during the unbinding process with CV1 value of 12.54 Å and 10.95 Å, respectively. The chain A and chain B of PD-L1 dimers are shown in green and cyan surface, respectively. The key residues (green or cyan) and ligands (orange) are shown in sticks. **(C,D)** The distance between Ile54, Tyr56, Met115, Ala121, and Tyr123 on conformation A and conformation B of PD-L1 during the unbinding process of BMS-8 and BMS-1166. **(E,F)** The extracted conformational state (green cartoon) of each complex with the largest distance between two PD-L1 monomers was overlapped with the original crystal structures (magenta cartoon).

From the free energy estimation of different conformational states, it can be seen that the conformational states of the crystal structures were much more stable than the other transition conformational states along the unbinding process. Therefore, the dissociation of small-molecule ligands of the initial conformational states tended to be the most important intermediate process for the unbinding of small-molecule ligands, which were corresponding to the minima of CV1 at 12.54 Å in BMS-8 dimer systems and the minima of CV1 at 10.95 Å in BMS-202 dimer systems. The corresponding transition states were extracted from the trajectories as shown in Figures 7A,B. The binding poses of BMS small-molecule ligands at the transition states were quite distinct from each other, which was probably owing to the difference of substituent groups. A common feature for both systems was that the ligands at transition states significantly lost the interaction with the chain A while the interaction with chain B were still compact and involved with a series of residues especially in BMS-1166 dimer systems. During the unbinding process, the core scaffold of ligands gradually divorced from the location of _A_Tyr56 and got away from the pocket formed by PD-L1 dimer. In order to monitor the conformational change of the pocket formed by PD-L1 monomers, the distance between chain A and chain B were calculated by the distance between Ile54, Tyr56, Met115, Ala121, Tyr123 on each chain as shown in Figures 7C–F. The conformational fluctuation of PD-L1 monomers was reflected by the conformational change of the F-G loops on both PD-L1 monomers. It can be seen that the pockets in BMS-8 and BMS-1166 complex systems were quite stable with occasionally occurring conformational fluctuations. According to unbinding processes of BMS ligands, it can be seen that the dimer of PD-L1 had a large tendency to keep stable although accompanied with subtle conformational fluctuation of PD-L1 dimer.

**Figure 8:**
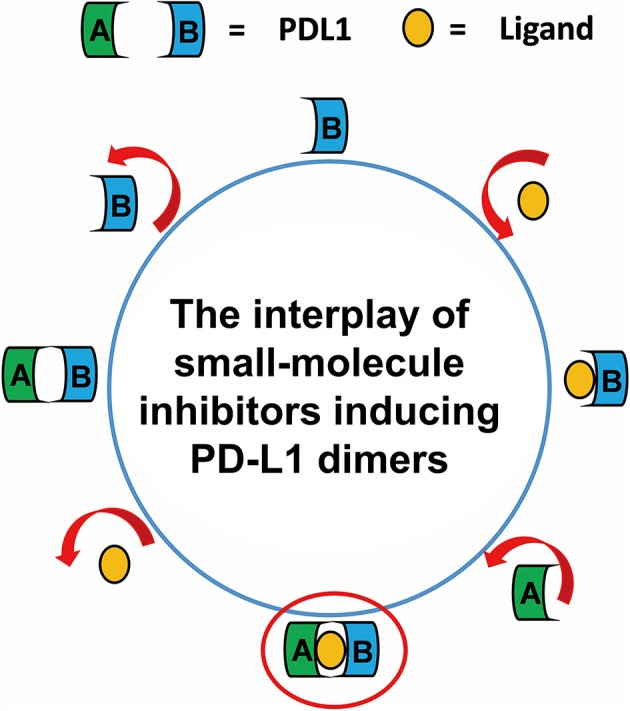
The complete binding and unbinding mechanism of BMS small-molecule inhibitors. The conformation A and conformation B of PD-L1 monomer, the small-molecule ligand are represented by green, light blue, orange objects, respectively.

Taken together, the most possible deduction for the interaction mechanism of BMS small-molecule inhibitors with PD-L1 was depicted as shown in Figure 8. Firstly, all BMS small-molecule inhibitors with different activities tended to interacted with a monomer conformation B of PD-L1. As the PD-L1 dimer complex had strong conformational stability, the PD-L1 monomer complex further interacted with the other monomer of PD-L1 to form PD-L1 dimer complex. According to the results of metadynamics simulation, a complete dissociation for BMS inhibitors would probably be like that the small-molecule ligand was firstly unbound from the PD-L1 dimer and the rest receptor part was further depolymerized into monomer.

### The R-Group QSAR Model of BMS Small-Molecule Inhibitors

**Figure 9:**
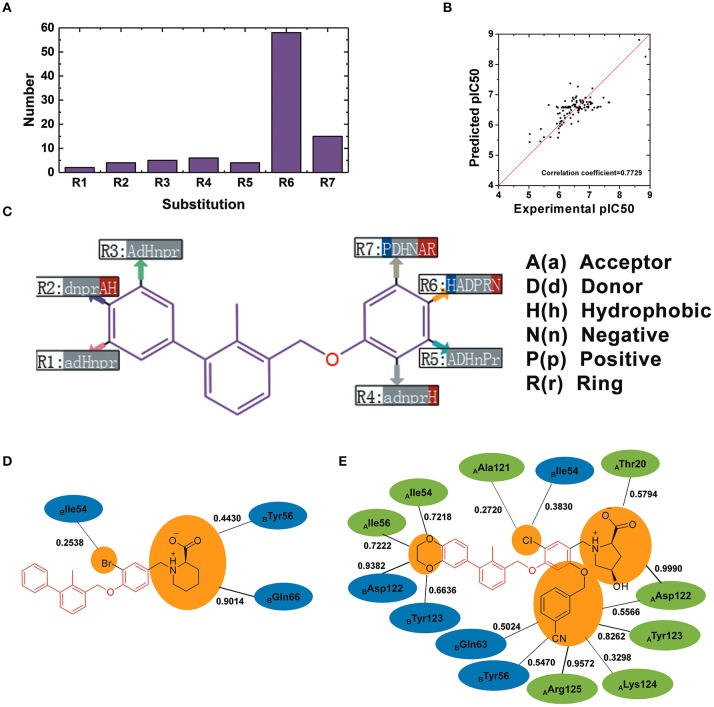
The R-group based QSAR for BMS small-molecule inhibitors. **(A)** The number of substituent groups at seven attachment points of BMS small molecule inhibitors. **(B)** The correlation validation between the predicted pIC50 and experimental pIC50. **(C)** R-group QSAR model for the BMS small molecule inhibitors. The case-insensitive alphabets A(a), D(d), H(h), N(n), P(p), and R(r), respectively represent the hydrogen bond acceptor, the hydrogen bond donor, the hydrophobic, the negative ionic, the positive ionic, aromatic ring. The significantly increase effect is colored in red while the significantly decrease effect is colored in blue. **(D,E)** The interacting residues of different substituent groups of BMS-8 and BMS-1166. The occupancies of the contact between each residues and substituent group were listed along with the black solid.

110 BMS small-molecule inhibitors with 2-methyl-3-(phenoxymethyl)-1,1′-biphenyl scaffolds were tested with diverse inhibitory activities with IC_50_ ranging from 9.492 μM to 1.4 nM. As shown in Figure 9A, there were 7 different of attachment points from R1 to R7 and the substituent groups of R6 and R7 had a relatively larger proportion than other attachment points. As shown in Figure 9B, the correlation coefficient between the predicted pIC_50_ and the experimental pIC_50_ was 0.7729. According to the evaluation of six key pharmacophore elements in Figure 9C, the substituent groups at R2, R4, R6, and R7 had obvious effect on the affinity of BMS small-molecule inhibitors. The substituent groups at R2, R4, R6, R7 of BMS-8 and BMS-116 as well as the interaction residues were recognized by the contact analysis as shown in Figures 9D,E. The contact analysis of BMS-1166 showed that the 1,4-benzodioxinyl group at R2 mainly was involved in the interaction with _A_Ile54, _A_Tyr56, _B_Asp122, _B_Tyr123. The hydrogen bond acceptor and hydrophobic groups at R2 were favorable for BMS inhibitors such as the 2, 3-dihydro-1, 4-benzodioxinyl group on BMS-114, BMS-200, BMS-1001, and BMS-1166. The analysis of effect of solvent in dimer systems showed that the substituent groups at R4, R5, R6, and R7 were exposed to solvent environment. The hydrophobic groups at R4 were favorable for BMS inhibitors, which corresponded to the fact that the bromine atom on BMS-8 and the chlorine atom on BMS-1166 had a close contact with _B_Ile54. The hydrophobic group at R6 was adverse while the negative ionic group was favorable. The substituent group at R6 of BMS-8 mainly interacted with _B_Tyr56 and _B_Gln66, however, that of BMS-1166 mainly interacted with _A_Thr20 and _A_Asp122. Nevertheless, the substituent group at R6 of BMS-8 and BMS-1166 both formed hydrogen bonding with PD-L1. The positive ionic at R7 was adverse while the hydrogen bond acceptor and aromatic ring were favorable. The substituent group at R7 of BMS-1166 formed interaction with _A_Asp122, _A_Tyr123, _A_Lys124, _A_Arg125, _B_Tyr56, and _B_Gln63. The comparison of the contact residues between BMS-8 and BMS-1166 indicated that the substituent group at R2 and R7 strongly strengthened the interactions with the conformation A of PD-L1, which was consistent with the stability of the monomer complex of conformation A and BMS-1166.

The further molecular docking study of eight representative small-molecule inhibitors showed that the docking scores had a good linear correlation with the experimental inhibitory activities (Figure S5). The further residue contribution comparison (Figure S6) and conformational analysis (Figure S7) of the residues within 5 angstroms showed that the residues interacting with R-group substituents had an obvious effect on the docking scores including _B_Asp122 (interacting with R1 to R3) and _A_Asp122, _A_Lys124, _B_Tyr56, _B_Gln66 (interacting with R4 to R7). The binding mode analysis of novel series of [1,2,4]triazolo[4,3-a]pyridines designed by Qin et al. also revealed the retaining hydrophobic interaction with Tyr56, Met115, and Ala121 on both chain of PD-L1 and extra π-π stacking with the _B_Tyr56 and π-anion interactions with _A_Asp122 (Qin et al., 2019). These interacting modes of [1,2,4]triazolo[4,3-a]pyridines inhibitors were consistent with the binding mode analysis of eight representative small-molecule inhibitors. It's suggested that the structure-activity relationship analysis of BMS small-molecule inhibitors was applicable for the further structure modifications.

## Conclusions

In this study, we used multiple molecular modeling methods to study the detailed molecular mechanism of the interaction between BMS small-molecule inhibitors and PD-L1. A detailed mechanism of the interaction process between small-molecule inhibitors and PD-L1 was proposed and validated by molecular dynamics simulations.

The BMS small-molecule inhibitors tended to interact with one PD-L1 monomer first and further formed dimer with the other monomer for an advantage of stability. The results of binding free energy and water occupancy calculation revealed the key stability factors for ligand-induced PD-L1 dimers including the hydrophobic contribution of Ile54, Tyr56, Met115, Ala121, and Tyr123 on both monomers and the water bridges involved in _A_Lys124. The unbinding pathway prediction also indicated that the tunnel formed by PD-L1 dimers tended to be stable upon the getting away of BMS-inhibitors. The R-group QSAR model suggested that the substituents at R2, R4, R6, and R7 had a significant effect on the inhibition activities of BMS inhibitors. The structural modification with these substituent positions tended to be an effective way to improve the inhibition activities of BMS inhibitors. Taken together, this study would provide a comprehensive view of the inhibition mechanism for BMS small-molecule inhibitors and guide the further development of more potential small-molecule inhibitors targeting PD-L1.

## Data Availability Statement

The raw data supporting the conclusions of this manuscript will be made available by the authors, without undue reservation, to any qualified researcher.

## Author Contributions

DS, HL, and XY designed the research. DS and XY were responsible for the writing and revising of the manuscript. XA, QB, SZ, and ZB were responsible the main data analysis in the manuscript.

### Conflict of Interest

The authors declare that the research was conducted in the absence of any commercial or financial relationships that could be construed as a potential conflict of interest.

## Supplementary Material

Figure S1 The interactions diagrams between the monomer conformation A of PD-L1 (the initial crystal structure and three respective dynamics structures) and BMS-8 in the monomer system of replica 2.

Figure S2 The interaction diagrams between the monomer conformation B of PD-L1 (the initial crystal structure and three respective dynamics structures) and BMS-8 in the monomer system of replica 2.

Figure S3 The interaction diagrams between the monomer conformation A of PD-L1 (the initial crystal structure and three respective dynamics structures) and BMS-1166 in the monomer system of replica 2.

Figure S4 The interaction diagrams between the monomer conformation B of PD-L1 (the initial crystal structure and three respective dynamics structures) and BMS-1166 in the monomer system of replica 2.

Figure S5 The linear correlation between experimental pIC50 and the absolute values of the docking scores.

Figure S6 The distance and residue contribution analysis of the binding poses of eight representative small-molecule inhibitors. **(A)** The respective and average distance between the small-molecule inhibitor and the residues on PD-L1 dimer. **(B)** The respective energy contribution of residues on PD-L1 dimer when interacting with the small-molecule inhibitor.

Figure S7 The binding pose analysis of eight representative small-molecule inhibitors. **(A)** The surrounding residues of the substituent groups at R1 to R3 for eight representative small-molecule inhibitors. **(B–I)** The surrounding residues of the substituent groups at R4 to R7 for small-molecule inhibitor with NO. of 4, 101, 102, 103, 104, 108, 119, 110, respectively.

Table S1 The detailed structural and activity information for 110 BMS small-molecule inhibitors.
