# Supplementary figures and images for: Computational Insight Into the Small Molecule Intervening PD-L1 Dimerization and the Potential Structure-Activity Relationship
Source: Front Chem. 2019 Nov 12;7:764. doi: 10.3389/fchem.2019.00764 (PMC6861162; doi:10.3389/fchem.2019.00764)

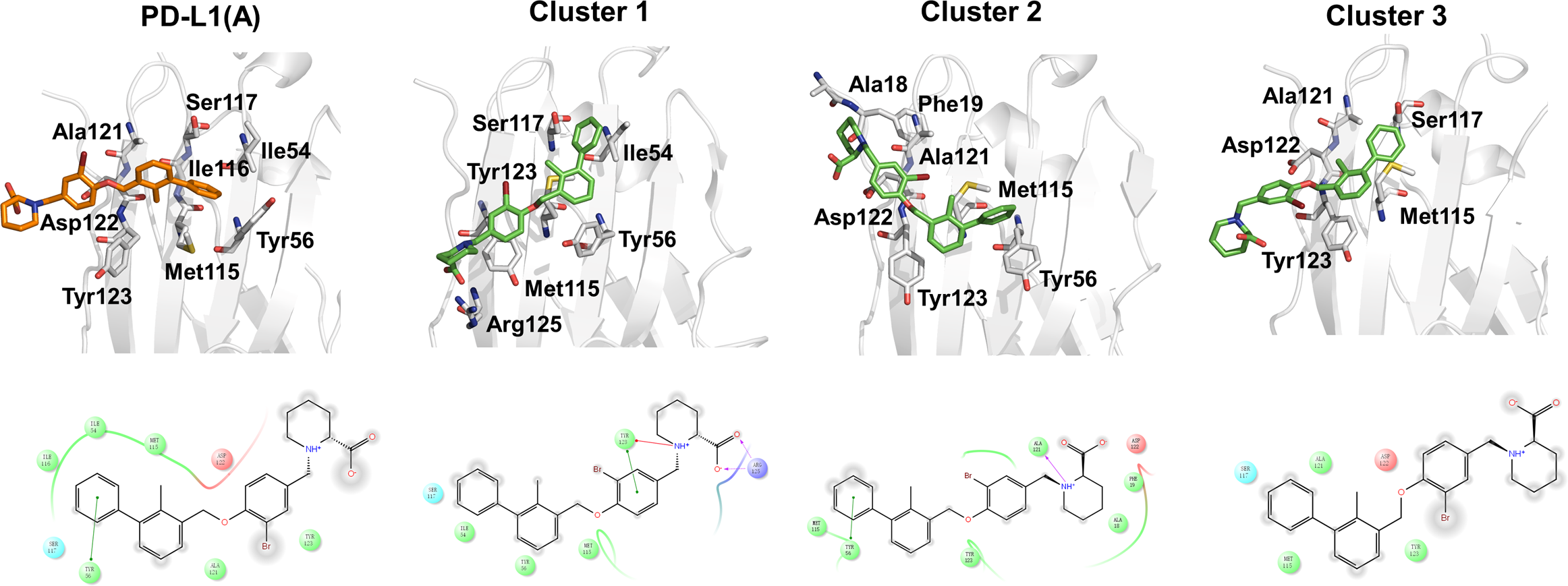

Supplement: Figure S1 — The interactions diagrams between the monomer conformation A of PD-L1 (the initial crystal structure and three respective dynamics structures) and BMS-8 in the monomer system of replica 2. [file Image_1.TIF]

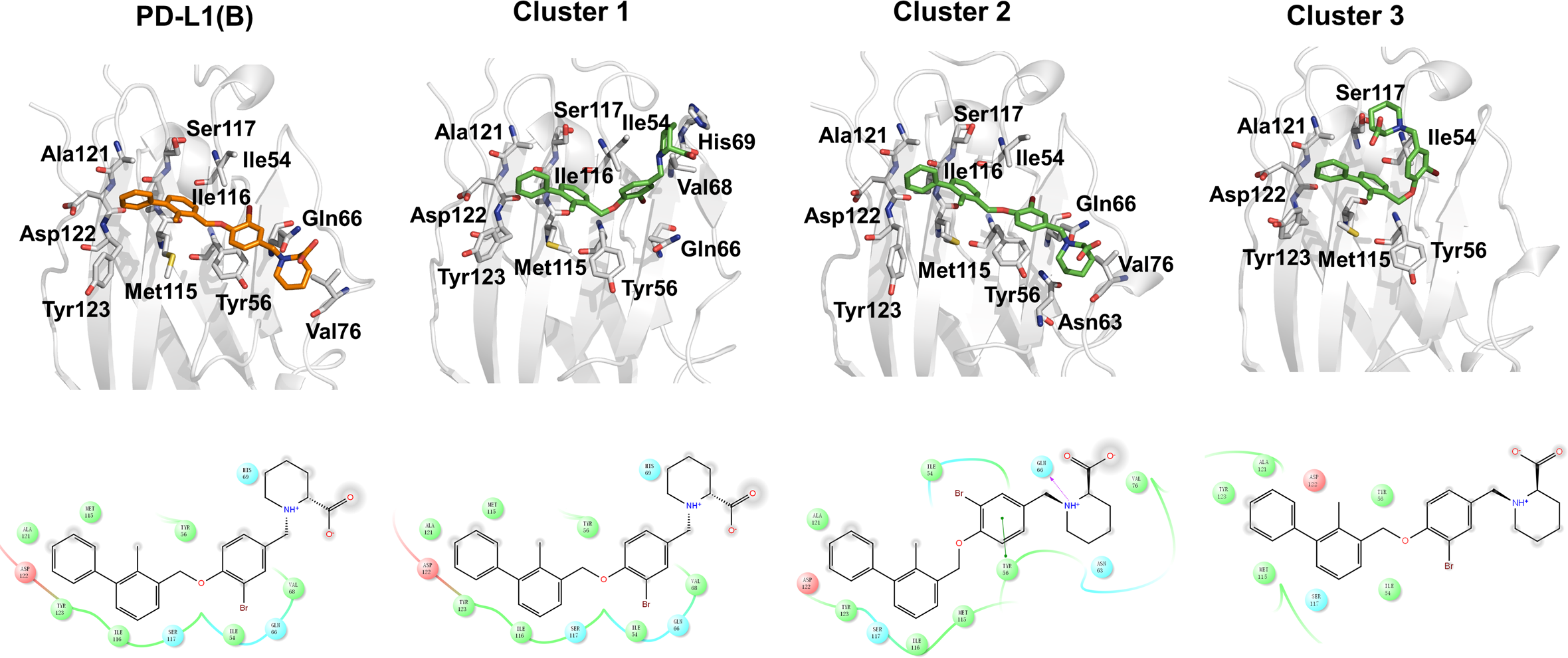

Supplement: Figure S2 — The interaction diagrams between the monomer conformation B of PD-L1 (the initial crystal structure and three respective dynamics structures) and BMS-8 in the monomer system of replica 2. [file Image_2.TIF]

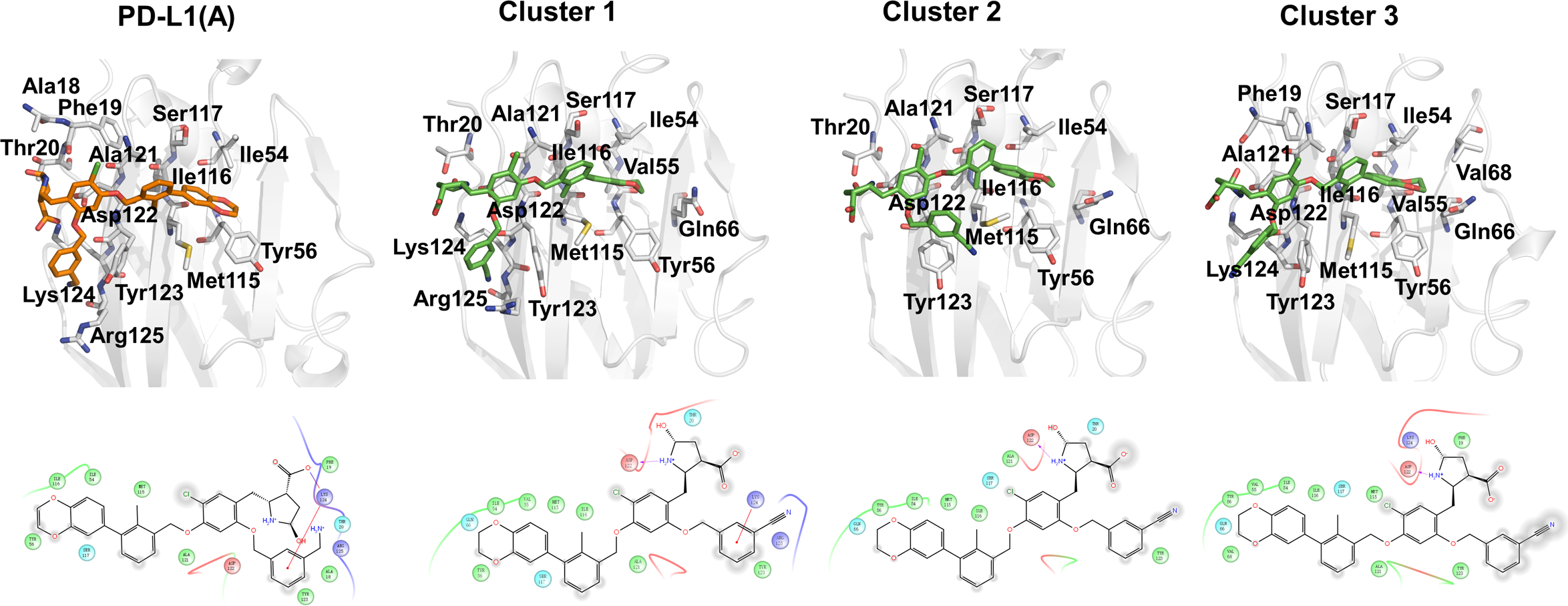

Supplement: Figure S3 — The interaction diagrams between the monomer conformation A of PD-L1 (the initial crystal structure and three respective dynamics structures) and BMS-1166 in the monomer system of replica 2. [file Image_3.TIF]

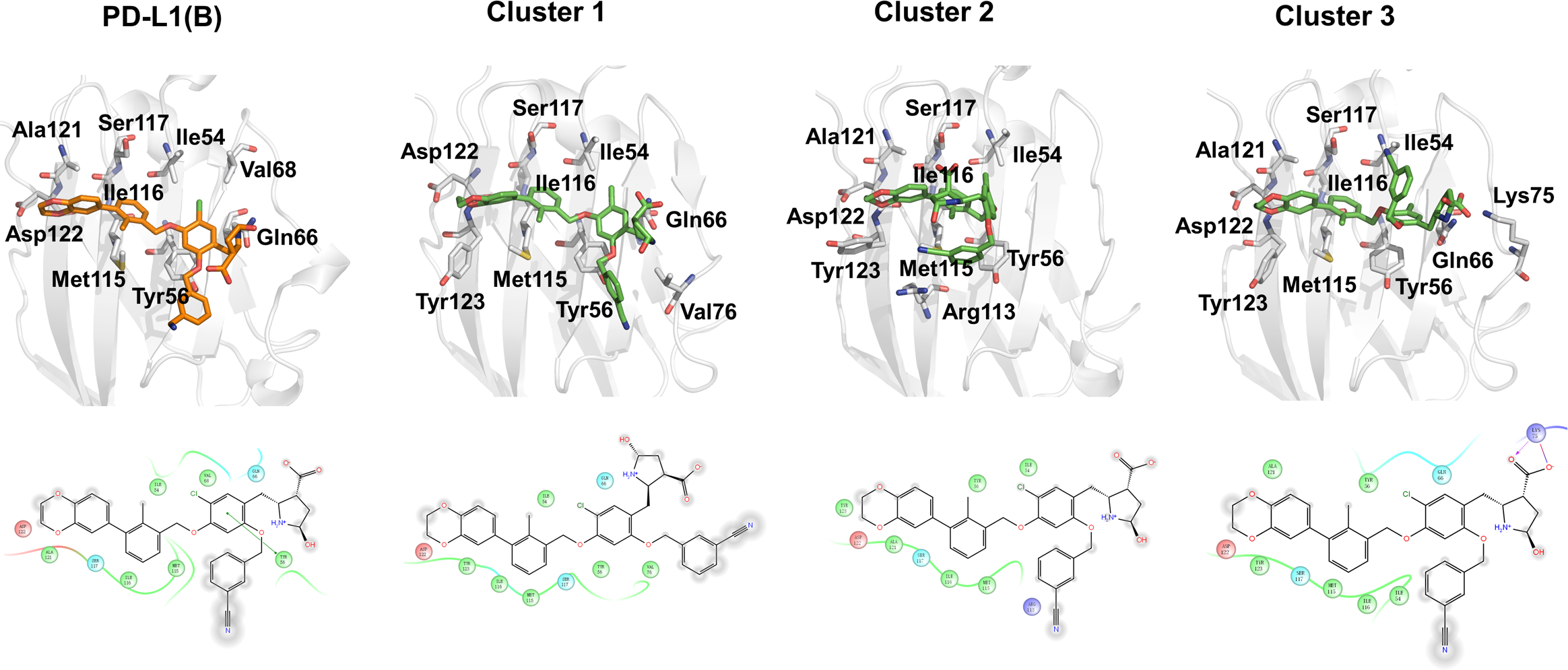

Supplement: Figure S4 — The interaction diagrams between the monomer conformation B of PD-L1 (the initial crystal structure and three respective dynamics structures) and BMS-1166 in the monomer system of replica 2. [file Image_4.TIF]

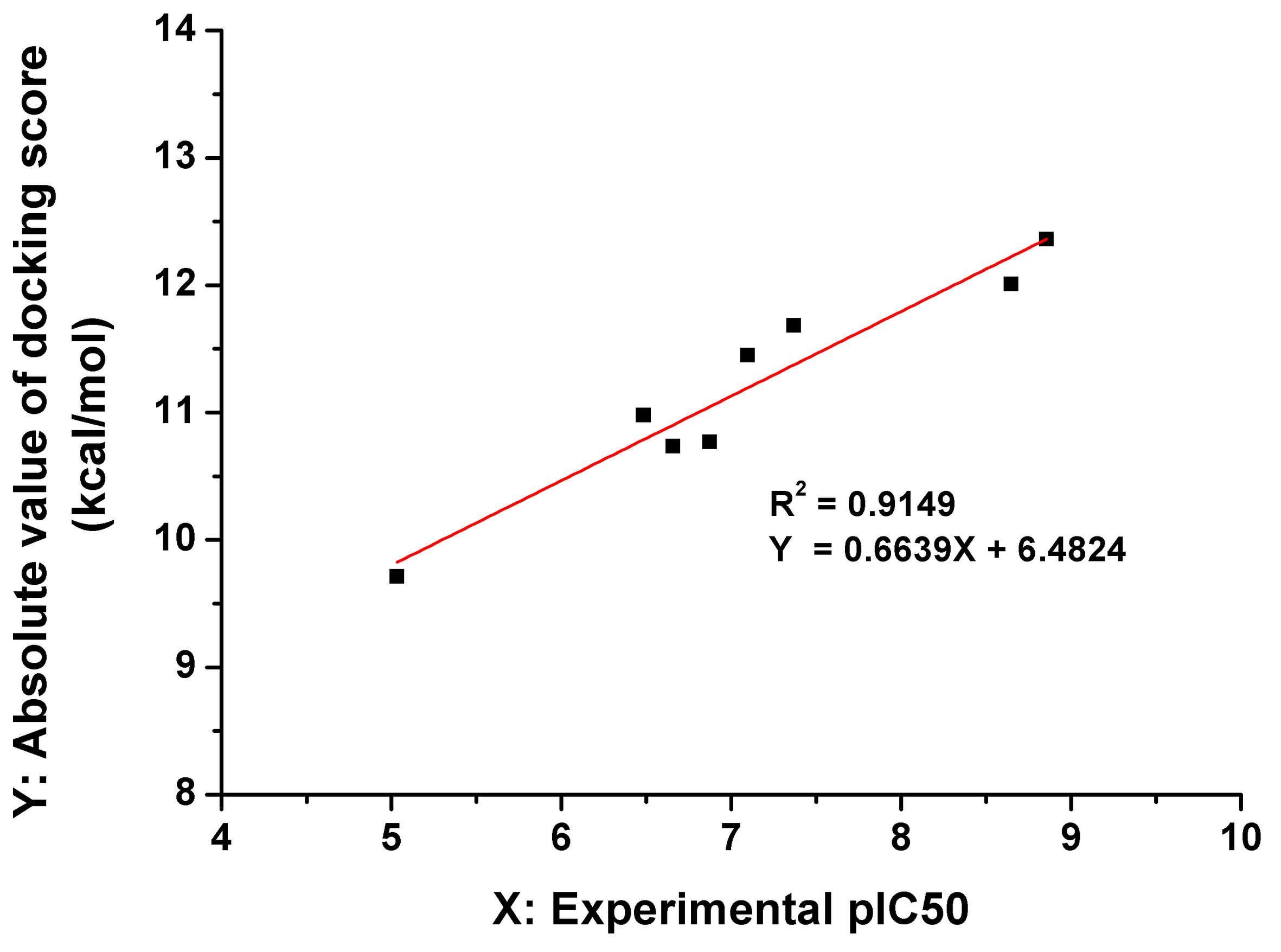

Supplement: Figure S5 — The linear correlation between experimental pIC50 and the absolute values of the docking scores. [file Image_5.TIF]

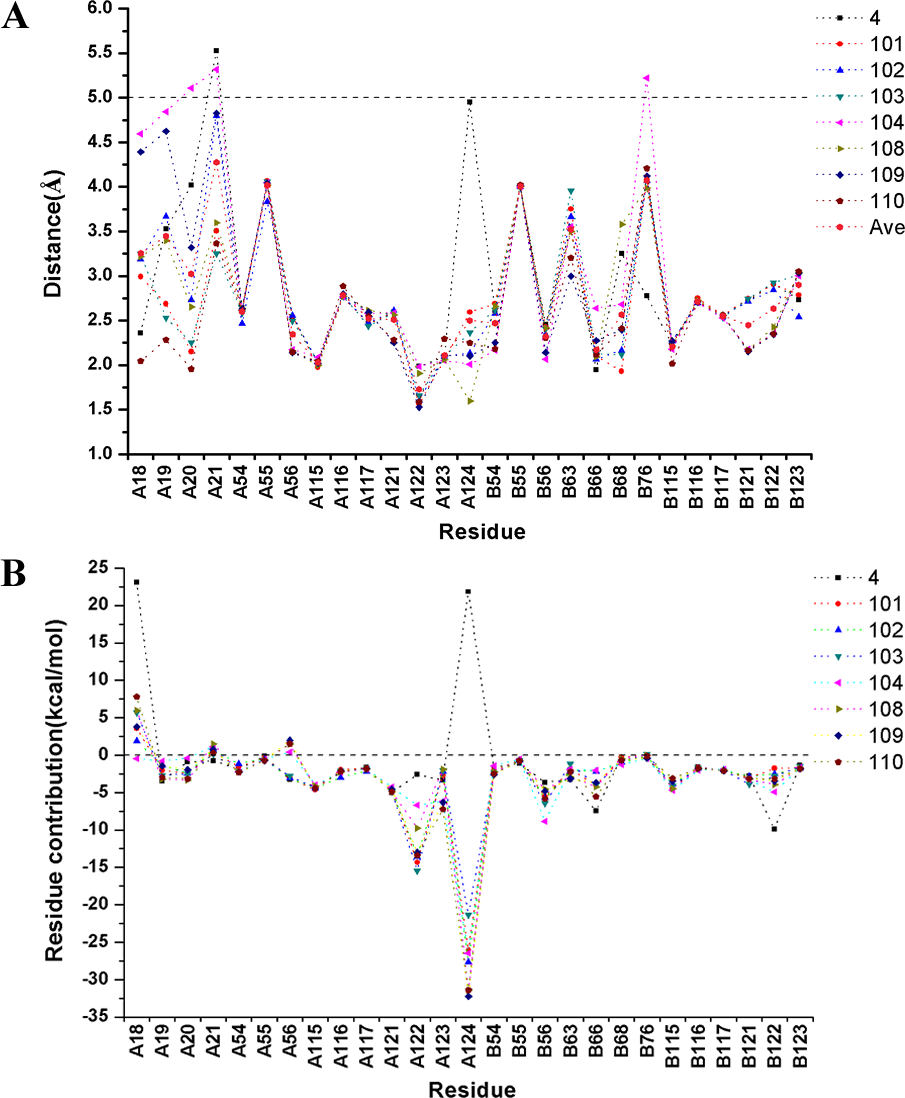

Supplement: Figure S6 — The distance and residue contribution analysis of the binding poses of eight representative small-molecule inhibitors. (A) The respective and average distance between the small-molecule inhibitor and the residues on PD-L1 dimer. (B) The respective energy contribution of residues on PD-L1 dimer when interacting with the small-molecule inhibitor. [file Image_6.tif]

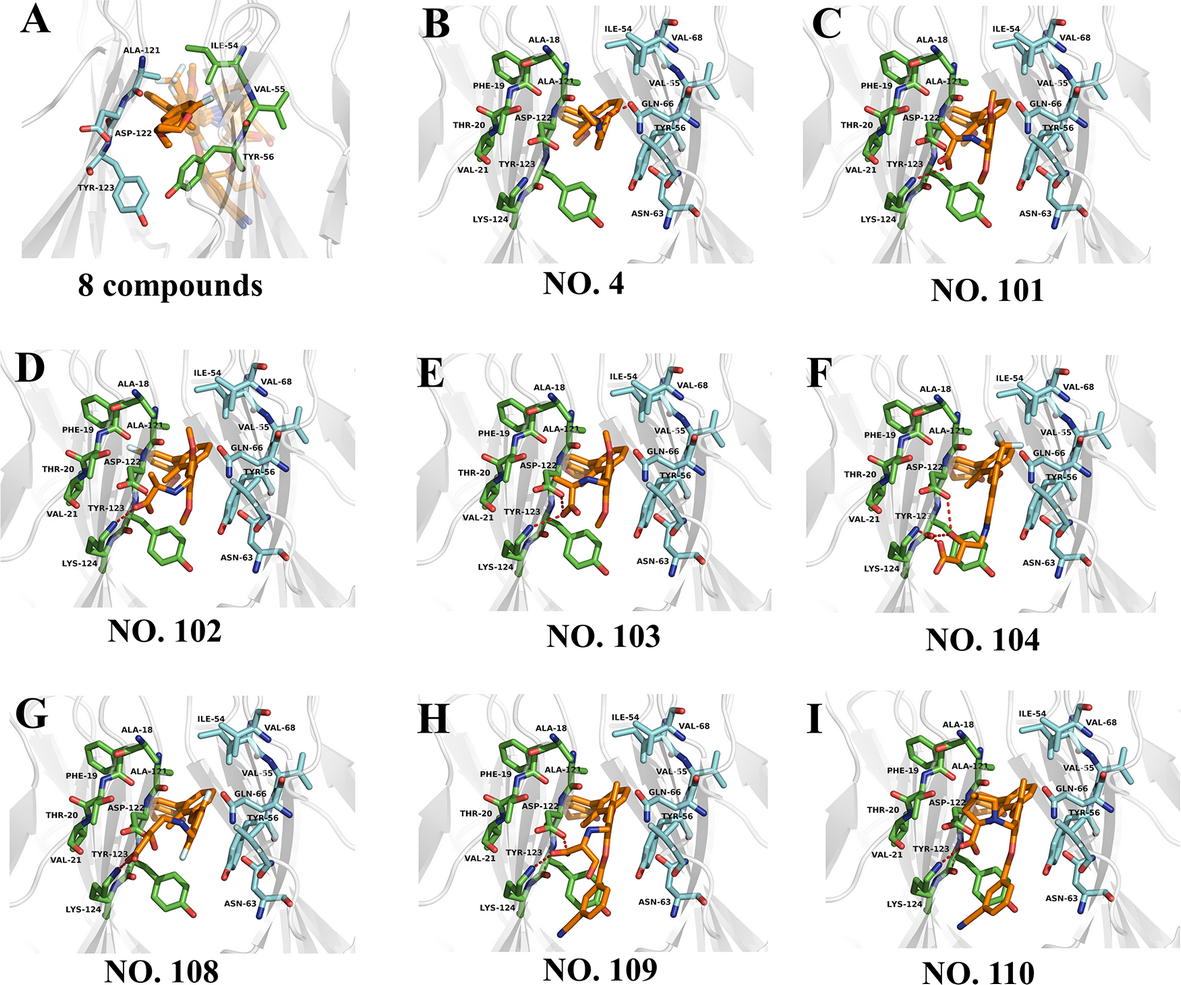

Supplement: Figure S7 — The binding pose analysis of eight representative small-molecule inhibitors. (A) The surrounding residues of the substituent groups at R1 to R3 for eight representative small-molecule inhibitors. (B–I) The surrounding residues of the substituent groups at R4 to R7 for small-molecule inhibitor with NO. of 4, 101, 102, 103, 104, 108, 119, 110, respectively. [file Image_7.tif]
