# Supplementary material for: Computational Insight Into the Small Molecule Intervening PD-L1 Dimerization and the Potential Structure-Activity Relationship
Source: Front Chem. 2019 Nov 12;7:764. doi: 10.3389/fchem.2019.00764 (PMC6861162; doi:10.3389/fchem.2019.00764)
Supplement: Table S1 — The detailed structural and activity information for 110 BMS small-molecule inhibitors. [file Table_1.DOCX]

**Table S1**. The detailed structural and activity information for 110 BMS small-molecule inhibitors.

| **NO.** | **Name** | **R1** | **R2** | **R3** | **R4** | **R5** | **R6** | **R7** | **Activity**  **(μM)** | **Experimental**  **pIC50** | **Predicted**  **pIC50** |
| --- | --- | --- | --- | --- | --- | --- | --- | --- | --- | --- | --- |
| 1 | BMS-1 | H | H | H | H | MeO | 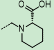 | MeO | 0.082 | 7.086 | 6.713 |
| 2 | BMS-2 | H | H | H | H | H | 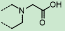 | \| 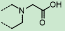 \| \| --- \| | 1 | 6.000 | 5.741 |
| 3 | BMS-3 | H | H | H | Cl | H | 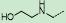 | H | 0.051 | 7.292 | 6.601 |
| 4 | BMS-4 | H | H | MeO | H | H | 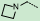 | H | 9.3 | 5.032 | 5.698 |
| 5 | BMS-5 | H | H | H | Br | MeO | \| 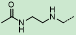 \| \| --- \| | MeO | 0.239 | 6.622 | 7.265 |
| 6 | BMS-6 | H | H | H | H | MeO | \| 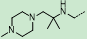 \| \| --- \| | MeO | 0.08 | 7.097 | 6.491 |
| 7 | BMS-7 | H | H | H | H | MeO | \| 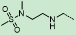 \| \| --- \| | MeO | 0.632 | 6.199 | 6.784 |
| 8 | BMS-8 | H | H | H | Br | H | \| 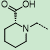 \| \| --- \| | H | 0.146 | 6.836 | 6.572 |
| 9 | BMS-9 | H | H | H | H | MeO | \| 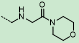 \| \| --- \| | MeO | 0.284 | 6.547 | 6.598 |
| 10 | BMS-10 | H | H | H | H | MeO | \| 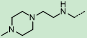 \| \| --- \| | MeO | 0.307 | 6.513 | 6.610 |
| 11 | BMS-11 | H | H | H | H | MeO | \| 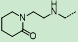 \| \| --- \| | MeO | 0.231 | 6.636 | 6.484 |
| 12 | BMS-12 | H | H | H | H | MeO | \| 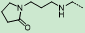 \| \| --- \| | MeO | 0.238 | 6.623 | 6.484 |
| 13 | BMS-13 | H | H | H | H | MeO | \| 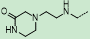 \| \| --- \| | MeO | 0.371 | 6.431 | 6.790 |
| 14 | BMS-14 | H | H | H | H | MeO | \| 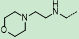 \| \| --- \| | MeO | 0.484 | 6.315 | 6.604 |
| 15 | BMS-15 | H | H | H | H | MeO | \| 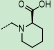 \| \| --- \| | MeO | 0.17 | 6.770 | 6.713 |
| 16 | BMS-16 | H | H | H | H | H | H | \| 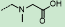 \| \| --- \| | 1.945 | 5.711 | 5.593 |
| 17 | BMS-17 | H | H | H | H | MeO | \| 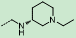 \| \| --- \| | MeO | 0.671 | 6.173 | 6.305 |
| 18 | BMS-18 | H | H | H | H | MeO | \| 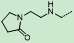 \| \| --- \| | MeO | 0.133 | 6.876 | 6.484 |
| 19 | BMS-19 | H | H | H | Me | H | \| 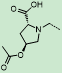 \| \| --- \| | H | 0.264 | 6.578 | 6.799 |
| 20 | BMS-20 | H | H | H | Me | H | \| 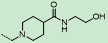 \| \| --- \| | H | 0.437 | 6.360 | 6.599 |
| 21 | BMS-21 | H | H | H | H | MeO | \| 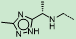 \| \| --- \| | MeO | 0.365 | 6.438 | 6.635 |
| 22 | BMS-22 | H | H | H | Br | H | 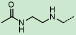 | H | 0.206 | 6.686 | 6.601 |
| 23 | BMS-23 | H | H | H | Cl | H | 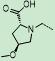 | H | 0.113 | 6.947 | 6.685 |
| 24 | BMS-24 | H | H | H | H | MeO | \| 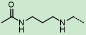 \| \| --- \| | MeO | 0.266 | 6.575 | 6.742 |
| 25 | BMS-25 | H | H | H | H | MeO | 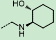 | MeO | 0.234 | 6.631 | 6.556 |
| 26 | BMS-26 | H | H | H | H | MeO | \| 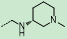 \| \| --- \| | MeO | 0.401 | 6.397 | 6.491 |
| 27 | BMS-27 | H | H | H | Me | MeO | \| 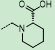 \| \| --- \| | H | 0.169 | 6.772 | 6.811 |
| 28 | BMS-28 | H | H | H | Me | H | \| 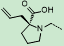 \| \| --- \| | H | 0.453 | 6.344 | 6.386 |
| 29 | BMS-29 | H | H | H | Br | H | \| 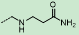 \| \| --- \| | H | 0.325 | 6.488 | 6.672 |
| 30 | BMS-30 | H | H | H | H | H | H | \| 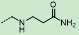 \| \| --- \| | 3.987 | 5.399 | 5.868 |
| 31 | BMS-31 | H | H | H | Cl | H | \| 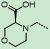 \| \| --- \| | H | 0.453 | 6.344 | 6.572 |
| 32 | BMS-32 | H | H | H | H | MeO | \| 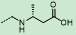 \| \| --- \| | MeO | 0.284 | 6.547 | 6.899 |
| 33 | BMS-33 | H | H | H | Cl | H | \| 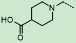 \| \| --- \| | H | 0.21 | 6.678 | 6.572 |
| 34 | BMS-34 | H | H | H | Cl | H | \| 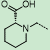 \| \| --- \| | H | 0.432 | 6.365 | 6.572 |
| 35 | BMS-35 | H | H | H | Cl | H | \| 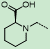 \| \| --- \| | H | 0.055 | 7.260 | 6.572 |
| 36 | BMS-36 | H | H | H | H | MeO | \| 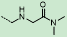 \|  \| \| --- \| --- \| | MeO | 0.292 | 6.535 | 6.670 |
| 37 | BMS-37 | H | H | H | H | MeO | \| 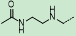 \| \| --- \| | MeO | 0.091 | 7.041 | 6.742 |
| 38 | BMS-38 | H | H | H | H | H | \| 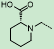 \| \| --- \| | MeO | 0.74 | 6.131 | 6.473 |
| 39 | BMS-39 | H | H | H | H | H | H | 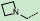 | 4.184 | 5.378 | 5.455 |
| 40 | BMS-40 | H | H | H | Me | H | \| 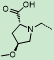 \| \| --- \| | H | 0.09 | 7.046 | 6.685 |
| 41 | BMS-41 | H | H | H | H | Me | \| 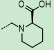 \| \| --- \| | Me | 0.815 | 6.089 | 6.008 |
| 42 | BMS-42 | H | H | H | Me | H | \| 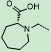 \| \| --- \| | H | 0.255 | 6.593 | 6.572 |
| 43 | BMS-43 | H | H | H | Me | H | \| 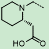 \| \| --- \| | H | 0.434 | 6.363 | 6.572 |
| 44 | BMS-44 | H | H | H | Me | H | \| 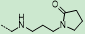 \| \| --- \| | H | 0.343 | 6.465 | 6.343 |
| 45 | BMS-45 | H | H | H | Cl | H | \| 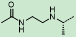 \| \| --- \| | H | 0.615 | 6.211 | 6.601 |
| 46 | BMS-46 | H | H | H | H | MeO | \| 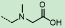 \| \| --- \| | MeO | 0.354 | 6.451 | 6.899 |
| 47 | BMS-47 | H | H | H | H | MeO | \| 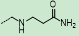 \| \| --- \| | MeO | 0.202 | 6.695 | 6.813 |
| 48 | BMS-48 | H | H | H | H | MeO | \| 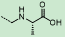 \| \| --- \| | MeO | 0.135 | 5.023 | 5.433 |
| 49 | BMS-49 | H | H | H | H | H | H | \| 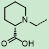 \| \| --- \| | 9.492 | 6.260 | 6.572 |
| 50 | BMS-50 | H | H | H | F | H | \| 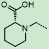 \| \| --- \| | H | 0.549 | 6.573 | 6.942 |
| 51 | BMS-51 | H | H | H | Me | H | \| 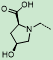 \| \| --- \| | H | 0.267 | 6.159 | 6.572 |
| 52 | BMS-54 | H | H | H | Me | H | \| 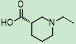 \| \| --- \| | H | 0.693 | 6.089 | 6.572 |
| 53 | BMS-55 | H | H | H | Me | H | \| 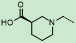 \| \| --- \| | H | 0.814 | 6.022 | 6.386 |
| 54 | BMS-56 | H | H | H | Me | H | \| 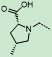 \| \| --- \| | H | 0.95 | 7.161 | 6.572 |
| 55 | BMS-57 | H | H | H | Me | H | 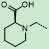 | \| H \| \| --- \| | 0.069 | 6.638 | 6.572 |
| 56 | BMS-58 | H | H | H | Me | H | \| 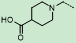 \| \| --- \| | H | 0.23 | 6.225 | 6.572 |
| 57 | BMS-59 | H | H | H | Me | H | \| 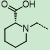 \| \| --- \| | H | 0.596 | 6.032 | 5.957 |
| 58 | BMS-60 | H | H | H | Me | H | H | \| 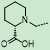 \| \| --- \| | 0.928 | 5.926 | 6.039 |
| 59 | BMS-61 | H | H | H | H | H | H | \| 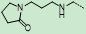 \| \| --- \| | 1.186 | 5.983 | 5.883 |
| 60 | BMS-63 | H | H | H | H | \| 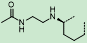 \| \| --- \| | 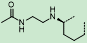 | H | 1.041 | 5.954 | 6.572 |
| 61 | BMS-64 | H | H | H | Me | H | \| 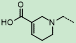 \| \| --- \| | H | 1.111 | 7.013 | 6.758 |
| 62 | BMS-65 | H | H | H | Cl | H | \| 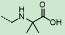 \| \| --- \| | H | 0.097 | 6.298 | 6.601 |
| 63 | BMS-66 | H | H | H | Cl | H | \| 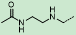 \|  \| \| --- \| --- \| | H | 0.503 | 6.379 | 6.230 |
| 64 | BMS-67 | H | H | H | Br | H | \| 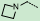 \| \| --- \| | H | 0.418 | 6.654 | 6.502 |
| 65 | BMS-68 | H | H | H | H | H | \| 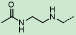 \| \| --- \| | MeO | 0.222 | 6.264 | 6.371 |
| 66 | BMS-69 | H | H | H | H | MeO | \| 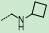 \| \| --- \| | MeO | 0.544 | 6.408 | 6.037 |
| 67 | BMS-70 | H | H | H | H | Me | \| 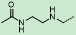 \| \| --- \| | Me | 0.391 | 6.121 | 6.601 |
| 68 | BMS-71 | H | H | H | Me | H | \| 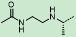 \| \| --- \| | H | 0.757 | 6.959 | 6.572 |
| 69 | BMS-72 | H | H | H | Me | H | \| 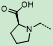 \| \| --- \| | H | 0.11 | 6.638 | 6.415 |
| 70 | BMS-74 | H | H | H | Me | H | \| 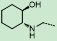 \| \| --- \| | H | 0.23 | 6.021 | 6.049 |
| 71 | BMS-75 | H | H | H | H | H | 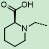 | \| H \| \| --- \| | 0.953 | 6.305 | 6.572 |
| 72 | BMS-76 | H | H | H | Me | H | 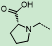 | \| H \| \| --- \| | 0.496 | 6.921 | 6.601 |
| 73 | BMS-77 | H | H | H | Me | H | \| 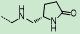 \| \| --- \| | H | 0.12 | 7.018 | 6.758 |
| 74 | BMS-78 | H | H | H | Cl | H | \| 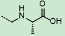 \| \| --- \| | H | 0.096 | 7.000 | 6.758 |
| 75 | BMS-79 | H | H | H | Cl | H | \| 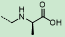 \| \| --- \| | H | 0.1 | 5.920 | 6.601 |
| 76 | BMS-80 | H | H | H | F | H | \| 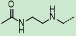 \| \| --- \| | H | 1.203 | 6.839 | 6.659 |
| 77 | BMS-81 | H | H | H | H | H | \| 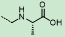 \| \| --- \| | MeO | 0.145 | 5.497 | 5.593 |
| 78 | BMS-82 | H | H | H | H | H | H | \| 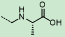 \| \| --- \| | 3.186 | 6.347 | 6.574 |
| 79 | BMS-83 | H | H | H | H | H | \| 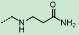 \| \| --- \| | MeO | 0.45 | 5.960 | 6.371 |
| 80 | BMS-84 | H | H | H | H | MeO | \| 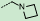 \| \| --- \| | MeO | 1.097 | 6.799 | 6.758 |
| 81 | BMS-85 | H | H | H | Me | H | \| 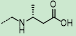 \| \| --- \| | H | 0.159 | 6.631 | 6.117 |
| 82 | BMS-86 | H | H | H | Me | H | \| 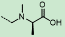 \| \| --- \| | H | 0.234 | 6.047 | 6.108 |
| 83 | BMS-87 | H | H | H | H | Me | \| 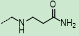 \| \| --- \| | Me | 0.898 | 6.750 | 6.529 |
| 84 | BMS-88 | H | H | H | Me | H | \| 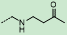 \| \| --- \| | H | 0.178 | 6.693 | 6.557 |
| 85 | BMS-89 | H | H | H | Me | H | H | \| 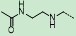 \| \| --- \| | 0.203 | 6.347 | 6.415 |
| 86 | BMS-90 | H | H | H | Me | H | \| 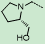 \| \| --- \| | H | 0.45 | 6.996 | 6.758 |
| 87 | BMS-91 | H | H | H | Me | H | \| 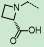 \| \| --- \| | H | 0.101 | 6.154 | 6.078 |
| 88 | BMS-93 | H | H | H | H | H | \| 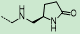 \| \| --- \| | H | 0.702 | 6.914 | 6.758 |
| 89 | BMS-94 | H | H | H | Me | H | \| 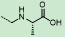 \| \| --- \| | H | 0.122 | 6.710 | 6.758 |
| 90 | BMS-95 | H | H | H | Me | H | \| 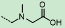 \| \| --- \| | H | 0.195 | 6.476 | 6.672 |
| 91 | BMS-96 | H | H | H | Me | H | \| 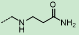 \| \| --- \| | H | 0.334 | 6.575 | 6.758 |
| 92 | BMS-97 | H | H | H | Me | H | \| 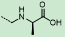 \| \| --- \| | H | 0.266 | 6.048 | 6.230 |
| 93 | BMS-98 | H | H | H | Cl | H | \| 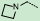 \| \| --- \| | H | 0.895 | 6.086 | 6.132 |
| 94 | BMS-99 | H | H | H | H | H | \| 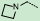 \| \| --- \| | MeO | 0.82 | 6.191 | 6.230 |
| 95 | BMS-100 | H | H | H | Me | H | \| 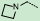 \| \| --- \| | H | 0.644 | 5.968 | 5.581 |
| 96 | BMS-101 | H | H | H | H | H | \| 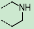 \| \| --- \| | 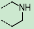 | 1.076 | 7.367 | 6.601 |
| 97 | BMS-102 | H | H | H | Me | H | \| 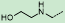 \| \| --- \| | H | 0.043 | 7.638 | 6.742 |
| 98 | BMS-103 | H | H | H | H | MeO | \|  \| \| --- \| | MeO | 0.023 | 6.710 | 6.758 |
| 99 | BMS-104 | H | H | H | Br | H | \|  \| \| --- \| | H | 0.195 | 7.469 | 6.899 |
| 100 | BMS-105 | H | H | H | H | MeO | \|  \| \| --- \| | MeO | 0.034 | 6.870 | 6.899 |
| 101 | BMS-106 | H | H | MeO | H | MeO | \|  \| \| --- \| | MeO | 0.134 | 6.873 | 6.890 |
| 102 | BMS-107 | F | H | MeO | H | MeO | \|  \| \| --- \| | MeO | 0.329 | 6.483 | 6.846 |
| 103 | BMS-108 | H |  | | H | MeO | \|  \| \| --- \| | MeO | 0.221 | 5.656 | 6.656 |
| 104 | BMS-114 | H |  | |  | H | H | \|  \| \| --- \| | 0.043 | 6.367 | 7.367 |
| 105 | BMS-163 | H | H | H | H | MeO | \|  \| \| --- \| | MeO | 0.093 | 7.032 | 6.904 |
| 106 | BMS-172 | H | H | H | H | MeO | \|  \| \| --- \| | MeO | 0.107 | 6.971 | 6.604 |
| 107 | BMS-174 | H | H | H | H | MeO | \|  \| \| --- \| | MeO | 0.022 | 7.658 | 6.742 |
| 108 | BMS-200 | H |  |  | F | H | \|  \| \| --- \| | F | 0.08 | 7.097 | 7.207 |
| 109 | BMS-1001 | H |  |  | Me | H | \|  \| \| --- \| |  | 0.00225 | 8.648 | 8.809 |
| 110 | BMS-1166 | H |  |  | Me | H | \|  \| \| --- \| |  | 0.0014 | 8.854 | 8.252 |
